# The End of “One Size Fits All” Sepsis Therapies: Toward an Individualized Approach

**DOI:** 10.3390/biomedicines10092260

**Published:** 2022-09-12

**Authors:** Jean-Louis Vincent, Tom van der Poll, John C. Marshall

**Affiliations:** 1 Department of Intensive Care, Erasme Hospital, Université Libre de Bruxelles, 1070 Brussels, Belgium; 2 Center of Experimental and Molecular Medicine, Amsterdam University Medical Centers, Location Academic Medical Center, University of Amsterdam, 1105 AZ Amsterdam, The Netherlands; 3 Division of Infectious Diseases, Amsterdam University Medical Centers, Location Academic Medical Center, University of Amsterdam, 1105 AZ Amsterdam, The Netherlands; 4 Departments of Surgery and Critical Care Medicine, St. Michael’s Hospital, Toronto, ON M5B 1W8, Canada

**Keywords:** biomarker, host response, personalized medicine

## Abstract

Sepsis, defined as life-threatening organ dysfunction caused by a dysregulated host response to an infection, remains a major challenge for clinicians and trialists. Despite decades of research and multiple randomized clinical trials, a specific therapeutic for sepsis is not available. The evaluation of therapeutics targeting components of host response anomalies in patients with sepsis has been complicated by the inability to identify those in this very heterogeneous population who are more likely to benefit from a specific intervention. Additionally, multiple and diverse host response aberrations often co-exist in sepsis, and knowledge of which dysregulated biological organ system or pathway drives sepsis-induced pathology in an individual patient is limited, further complicating the development of effective therapies. Here, we discuss the drawbacks of previous attempts to develop sepsis therapeutics and delineate a future wherein interventions will be based on the host response profile of a patient.

## 1. Introduction

The word “sepsis” (σήψη) comes from an ancient Greek word meaning putrefaction. It described the consequence, and not the cause, of a process. With the identification of microorganisms as the vectors of infection in the 19th century, the word “sepsis” was seconded to describe the clinical disease associated with severe infection. Terminology evolved further to consider the condition as a clinical syndrome called the ”sepsis syndrome” [1]. This approach provided a convenient means of identifying a patient who was likely to have a serious infection, and so might benefit not only from antibiotics, and possibly source control, but also fluid repletion and organ support, and admission to an intensive care unit (ICU).

The inflammatory response associated with sepsis, characterized by fever, abnormal white blood cell count, and increased concentrations of inflammatory markers such as C-reactive protein, suggested a role for the administration of anti-inflammatory agents as therapies, but beneficial effects on outcomes with this strategy were limited. As the pathophysiology of sepsis has become better understood, and with increased awareness of the concomitant presence of an anti-inflammatory response, sepsis has come to be considered a ”dysregulated host response” [2]. Multiple attempts to control, limit, or augment individual components of the immune response have been evaluated, but with little success in the clinical setting. Even strategies targeting pathological processes common to most patients with sepsis, including microvascular dysfunction associated with endothelial cell dysfunction and coagulopathy, have shown little evidence of benefit.

Here, we will briefly review the different approaches to modifying the host response to sepsis and consider what lies ahead.

## 2. The ”Unbalanced” Immune Response of Sepsis

A balanced immune response to an infection is localized and characterized by concurrent inflammatory, anti-inflammatory, and reparative reactions, with pathogen eradication and return to physiological homeostasis [3]. Sepsis represents an unbalanced immune response, wherein the causative microorganism has surpassed the protection provided by an adequate immune response, and the growing bacterial load continues to stimulate host cells, causing injury and a failure to return to homeostasis [3]. Notably, in sepsis, many of the immune mechanisms originally activated to organize a balanced response become detrimental, with features of excessive inflammation and immune suppression. Early attempts to modulate the aberrant immune reaction in sepsis focused on inhibition of hyperinflammation. In particular, proinflammatory cytokines, such as tumor necrosis factor (TNF) and interleukin (IL)-1β, were considered to play major roles in the so-called cytokine storm in acute sepsis. This assumption was primarily based on preclinical models in which high doses of bacteria or bacterial products were administered systemically to animals; blocking TNF or IL-1 conferred strong protective effects in such models [4]. However, strategies targeting individual inflammatory mediators in clinical trials, for example using anti-TNF antibodies [5], IL-1 receptor antagonists (IL-1ra) [6], or antagonists to platelet-activating factor (PAF) [7] yielded disappointing results. Unrestrained activity of proinflammatory cytokines is still considered to be involved in injury in sepsis, but there is now a consensus that systemic challenge models do not resemble human sepsis.

Neutrophils play a role in the hyperinflammation in sepsis through the secretion of proteases and reactive oxygen species. Moreover, neutrophils can release neutrophil extracellular traps (NETs), containing chromatin, antimicrobial peptides, and proteases, which, on the one hand, are important for antibacterial defense mechanisms, but on the other hand, can cause harm through various mechanisms, including initiation of intravascular thrombosis and multiple organ failure [8].

Sepsis is also associated with the activation of the coagulation and complement systems, between which, close interactions exist. Whilst complement activation is key to protective innate immunity, unrestrained activation can damage tissues and induce organ failure [9]. Likewise, components of the coagulation system can trigger protective innate defense mechanisms; yet, in sepsis, activation of the coagulation system becomes unbalanced, causing an increased tendency to microvascular thrombosis [10]. Sepsis-induced coagulopathy can culminate in disseminated intravascular coagulation (DIC), which can be associated with thrombosis, and—due to consumption of clotting factors, anticoagulant proteins, and platelets—bleeding [10]. The main initiator of coagulation in sepsis is tissue factor, induced on endothelial cells, monocytes, and macrophages by microbial agents and inflammatory mediators, including cytokines and complement factors [10,11]. In sepsis, large amounts of tissue factor are present in microvesicles derived from several cellular sources, which can bind to other cells, thereby augmenting coagulation. Inhibition of tissue factor in humans and nonhuman primates strongly attenuated coagulation activation after infusion of lipopolysaccharide or bacteria [10]. The prothrombotic state in sepsis is intensified by impaired function of three main anticoagulant pathways, i.e., antithrombin, tissue factor pathway inhibitor (TFPI), and the protein C system [10]. Decreased expression of thrombomodulin, an endothelial cell receptor that catalyzes the production of activated protein C, has an important role in this process.

## 3. The Anti-Inflammatory Approach to Sepsis Treatment

Corticosteroids are potent and readily available anti-inflammatory agents and were among the first strategies to be proposed and evaluated in the treatment of sepsis. Early work evaluated the effects of large doses of dexamethasone or methylprednisolone, to maximize their anti-inflammatory role, in patients with septic shock [12,13,14]. The results overall were disappointing [13,14], although these early trials were conducted in relatively small patient populations. The next page in the corticosteroid story came with the suggestion that patients with sepsis may have “relative adrenal insufficiency”, and that lower “replacement” doses of corticosteroids may be effective [15]. The administration of hydrocortisone to patients with severe septic shock was associated with reduced 90-day mortality [16] and is recommended in recent guidelines [17]. Nonsteroidal anti-inflammatory agents, such as ibuprofen, have not proven beneficial, even when administered to combat fever [18].

Table 1 collates some of the most studied of these interventions targeting the hyperinflammation of sepsis.

There are several reasons for the lack of success with this approach:

1. Efficacy in clinical trials of sepsis therapies has generally been measured as increased 28-day survival. However, mortality in patients with sepsis can be determined by multiple factors, including the underlying disease, premorbid conditions and complications, adequacy of therapy, and patient preferences in terms of end-of-life management. Thus, only a small and unknown component of the risk of death in these patients can be prevented by an effective treatment. The assumption that absence of a positive effect on 28-day survival implies a lack of clinical efficacy has been a common shortcoming in clinical trials conducted in heterogeneous ICU populations [50].
2. The biologic response mediated by TNF and other proinflammatory substances is an evolutionarily conserved one that has enabled multicellular organisms to survive infection. Indeed, the essential role of TNF in antimicrobial defense has been documented in several preclinical studies, especially in the context of pneumonia, the most common source of sepsis [51,52]. Blocking this pathway may therefore negatively affect survival.
3. There are multiple, interacting, and redundant biologic pathways, such that blocking any one may not be sufficient, and indeed may be harmful.
4. The anti-inflammatory response actually occurs very early; for example, IL-10 is released very early in the process of sepsis [53] and may become predominant quite early in the process [54]. Thus, some patients may benefit from immunostimulation, rather than anti-inflammatory treatment.

## 4. Moving from Anti-Inflammatory Strategies to Immunostimulating Strategies?

As the consequence, in part, of failed clinical trials seeking to inhibit inflammation in sepsis patients, the attention of the scientific community has shifted towards immune suppressive alterations in more recent years. The immune suppression associated with sepsis primarily results from enhanced apoptotic death of immune cells, T-cell exhaustion, and reprogramming of antigen-presenting cells through epigenetic changes [55,56]. Interventions that target apoptosis exerted beneficial effects on several outcomes in preclinical sepsis models [55]. Lymphocytes from patients with sepsis, either from blood or spleen (derived from “fresh” postmortem material), had impaired capacity to produce cytokines [57,58,59]. Regulatory T cells and myeloid-derived suppressor cells further shape the anti-inflammatory setting in sepsis [59,60]. Myeloid-derived suppressor cells are a mixed group of mostly immature myeloid cells that inhibit effector immune cells, particularly T cells [61], and expansion of these cells is associated with a higher risk of secondary infections in patients with sepsis [62]. Neutrophil function can also be impaired in sepsis, including reduced chemotactic migration, intracellular granule content, and oxidative burst capacity [63].

In recent years, checkpoint regulators have been implicated in immune suppression in sepsis. Checkpoint regulators function as a second signal to orchestrate the immune response to an antigen [64]. Programmed cell death-1 (PD-1) is a checkpoint regulator that has been widely studied in the context of sepsis pathogenesis. Stimulation of T-cell PD-1 triggers the secretion of immunosuppressive molecules and may induce apoptosis. Increased T-cell PD-1 expression was associated with impaired T-cell proliferative capacity, greater occurrence of nosocomial infections, and a higher mortality in patients with sepsis [65]. Sepsis patients also demonstrated elevated expression of PD-1 on blood monocytes and granulocytes [66], PD-1 on T cells, and PD-L1 levels on antigen-presenting cells, associated with T-cell apoptosis, lymphocytopenia, and mortality [57,65,67]. The functional relevance of the PD-1 pathway is reinforced by studies reporting improved survival of mice with experimentally induced sepsis in which PD-1 was either blocked or genetically eliminated [68,69]. Consequently, these results have led to the proposition that PD-1/PD-L1 pathway inhibitors may reverse immune suppression in sepsis. Several small clinical trials with an anti-PD-L1 antibody have been performed in patients with sepsis, in whom anti-PD-L1 treatment induced an increase in absolute lymphocyte counts and monocyte HLA-DR expression [70] and was well tolerated [70,71,72].

Reprogramming of monocytes and macrophages, resulting in a reduced ability to produce proinflammatory cytokines upon ex vivo stimulation and a diminished expression of MHC class II at the cell surface, is a widely published phenomenon in sepsis [57,73,74,75]. Indeed, reduced expression of MHC-II on blood monocytes is considered a surrogate marker for sepsis-induced immunosuppression, correlating with adverse outcomes such as a higher occurrence of nosocomial infections and increased mortality [76,77]. The impaired capacity of blood leukocytes to mount proinflammatory responses upon restimulation may relate to deficiencies in the ability to activate the master regulator of inflammation, nuclear factor-κB, as indicated by intracellular flow cytometry analyses of ex vivo stimulated leukocyte subsets in blood of patients with sepsis [78]. Epigenetic changes, particularly through histone modifications and DNA methylation, play an important role in the reprogramming of immune cells in sepsis [3].

Sepsis-induced immune suppression has been linked to the increased vulnerability of patients to secondary infections, oftentimes caused by pathogens that are weakly virulent and/or opportunistic [75]. Similarly, patients with sepsis are more susceptible to acquiring systemic fungal infections, most prominently candidiasis, and relatively frequently, they display signs of reactivation of dormant viruses, such as of cytomegalovirus, herpes viruses, and Epstein–Barr virus infections.

**Figure 1:**
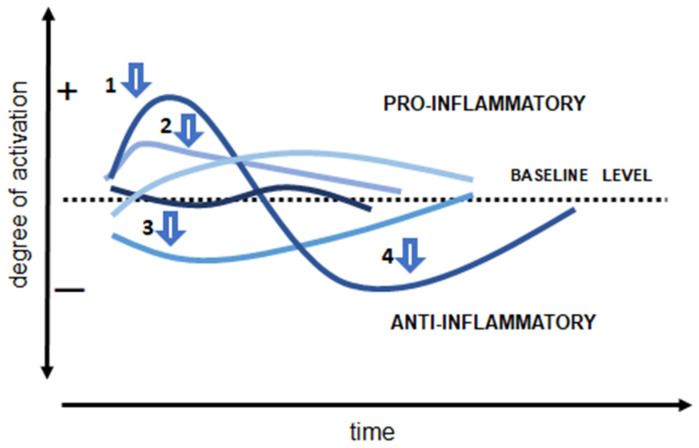
Each patient follows their own trajectory of immune response activation during the course of sepsis, rather than all following the same pattern. The early response is usually proinflammatory and the later response immunosuppressive, but there are exceptions, and the time course can vary substantially from one patient to the other. The arrows indicate some examples of time points at which an anti-inflammatory strategy may be beneficial (1 and perhaps 2) and others when it may be harmful (3 and 4).

The documentation of immune suppression in sepsis has triggered the design and execution of trials with immune-enhancing compounds in sepsis patients. This corollary approach—using immunostimulating agents, such as interferon-γ, granulocyte–macrophage-colony-stimulating factor (GM-CSF), IL-7, and anti-PD1 antibodies [75,79])—is likely to be as ineffective as the use of anti-inflammatory strategies if it is assumed that all patients react in the same way and follow the same immune response trajectory (Figure 1).

Moreover, although acquired relative immunosuppression may predispose to nosocomial infections, it may not substantially increase mortality [80].

## 5. Moving towards Personalized Strategies?

The heterogeneity evident among patients with sepsis, with variability in terms of age, comorbidities, infecting organism, genetic predisposition, suggests that a ”one size fits all” approach is unlikely to be successful. Applying a therapy to patients who are most likely to respond to it in a more individualized approach is intuitively attractive. In one case history, a patient with extensive mucormycosis, who was profoundly immunosuppressed, responded to immunostimulatory therapy combining interferon-γ with a PD-1 inhibitor [81]. However, how can different therapies be best matched to patients? Therapies could be guided by biomarkers, although there are three major limitations to this approach. First, not all cells are in an identical state at any one time; rather, proinflammatory and immunosuppressed responses may coexist [82]; thus, the interpretation of biomarker concentrations can be complex. Second, a patient’s condition can evolve more rapidly than changes in biomarkers, such that by the time altered biomarker levels are recognized, the situation may already have changed, and the suggested treatment may no longer be appropriate. Third, biomarkers are usually measured in the blood or bloody fluids, but the situation in the tissues may not be the same.

Several retrospective analyses have supported the concept of biomarker-guided selection of sepsis patients for specific immunomodulatory strategies. For example, a retrospective subgroup analysis of randomized controlled trial data suggested that stratification by baseline plasma IL-1ra concentrations resulted in more promising results, because those with high plasma IL-1ra concentrations appeared to benefit from recombinant IL-1ra therapy, whereas other patients did not [83]. An alternative approach is to identify different phenotypes, or endotypes, which could help to identify the best therapeutic options for individual patients [84]. In a post hoc analysis of a double-blind, randomized clinical trial, patients categorized as having a relatively immunocompetent phenotype were more likely to have higher mortality when treated with corticosteroids than patients with a relatively immunosuppressed phenotype [85]. Similarly, in a retrospective transcriptomic study, patients with bacterial sepsis could be categorized as having an “inflammopathic”, an “adaptive”, or a “coagulopathic” phenotype [86]. These phenotypes were associated with different clinical severity and outcomes and may potentially respond to different therapeutic approaches.

Some clinical trials have already attempted to enrich study populations with patients more likely to respond to the therapy under investigation. For example, in the MONARCHS trial [87], which tested the effects of an anti-TNF antibody in patients with sepsis, only patients with high baseline IL-6 levels—reflecting enhanced systemic inflammation—were included in the primary efficacy analysis. In the SCARLET trial [26], the platelet count and international normalized ratio were used to enrich the population with patients more likely to benefit from thrombomodulin administration. Several studies assessing the effects of immune stimulants have also attempted to enrich the population, by including only patients with immune suppression, as reflected by low expression of HLA-DR on circulating monocytes and/or low lymphocyte counts [71,73,88]. “Immunsep” is an ongoing clinical trial (NCT04990232) in which patients are stratified as having hyperinflammation or immune paralysis according to their ferritin and HLA-DR status and treated with IL-1ra or interferon-γ, respectively.

For the past four decades, going back to the first clinical trials of high-dose corticosteroids [13], sepsis clinical trials have used a common model, based on a series of common assumptions. It was assumed that patients who might benefit from interventions that targeted highly diverse components of a complex biologic response could be identified on the basis of arbitrary and nonspecific physiologic criteria, in conjunction with clinical evidence of infection and new-onset organ dysfunction. These assumptions have been reiterated in varying forms as consensus definitions were refined [2,89,90]. They have not, however, been fundamentally challenged: it is time to do so.

Sepsis is a complex disorder, and its therapy is multimodal. For some patients, aggressive antifungal therapy may be life-saving, for others, it may be futile or even harmful. Some patients benefit from emergent surgical intervention, though for most, surgery has no role. Fluid resuscitation or intubation and mechanical ventilation may help some patients but be unnecessary for others. Our challenge is not simply to determine whether most patients who meet generic sepsis criteria will benefit from a particular therapeutic strategy, but rather to determine in which patients, and at what stage of their illness, is such an approach most likely to confer benefit.

The phrase “precision medicine” first appeared in the biomedical literature at the end of the first decade of the 21st century [91,92]. Its emergence reflected the coalescence of several factors: the completion of the human genome project and an evolving awareness of intrinsic heterogeneity amongst individuals; emerging evidence of heterogeneity of treatment effect for treatments that targeted a single disease; and evidence that biologic heterogeneity in specific cancers—for example, expression of estrogen receptors or Her2/Neu in women with breast cancer—could be used to guide the use of therapies that targeted these tumor markers [93].

Oncology can provide useful insights to advance personalized medicine in the setting of critical illness. It is telling that oncologists do not convene regular consensus meetings to define cancer. The World Health Organization acknowledges the inherent imprecision of the word in its definition of cancer: “Cancer is a large group of diseases that can start in almost any organ or tissue of the body when abnormal cells grow uncontrollably, go beyond their usual boundaries to invade adjoining parts of the body and/or spread to other organs.” [94]. An analogous approach to sepsis would acknowledge that sepsis is not a single disease, but a descriptive term for a group of diseases that arise through a dysregulated host response to infection.

Beginning with the work of Pierre Denoix in the 1940s [95], cancer researchers sought to develop improved methods of staging and stratifying cancer, recognizing that cancers differed not only in the cell of origin, but also in the degree of spread at the time of initial diagnosis. The output of this exercise was the TNM (tumor, nodes, metastasis) model for staging cancer based on cell or tissue of origin, and extent of local, regional, and distal spread. The system is updated on a regular basis by the Union for International Cancer Control (UICC), and is now in its eighth iteration [96]. While these initial efforts to stage cancer were directed towards better prognostication, the TNM model shaped the basic approach to the multimodal treatment of a complex disease. Those cancers that are localized can be treated, and often cured by approaches that only target the primary cancer—surgery, and occasionally radiotherapy. Chemotherapy is reserved for cancers where there is either evidence of spread beyond the primary site at the time of diagnosis (typically microscopic deposits in regional lymph nodes), or for which the probability of recurrence is high. Thus, chemotherapy is targeted towards those patients most likely to benefit, avoiding the exposure of patients to toxic therapies that would not alter the natural history of their disease. Cancers that have spread more widely—those with distant metastases—are managed with more potent systemic therapies, including interventions that modify the intracellular pathways that support abnormal cell growth.

An early effort to reproduce the TNM model in acute illness proposed an analogous model based on Predisposition, Insult, Response, and Organ dysfunction—the PIRO model [90]. While it is clear that such an approach can segregate groups of patients on the basis of both prognosis and response to therapy [97], how best to achieve this is unclear, because acute illnesses such as sepsis lack histologic measures of extent, and the role of the hundreds of biomarkers of sepsis that have been described is unclear [98].

Oncology has moved beyond clinical staging with the recognition that oncogenic pathways and mechanisms may be shared amongst multiple tumor types. Immune checkpoint inhibitors, exemplified by inhibitors of PD-1/PD-L1 interactions, have been shown to be effective in the treatment of both advanced melanoma and non-small-cell lung cancer [99], and more recently, in a small but remarkable case series, to eliminate locally advanced rectal cancers associated with a deficiency in mismatch repair [100]. Applied to acute illness, these insights open the door not only to identifying abnormal biologic processes that can be targeted using specific molecular inhibitors, but also to using these approaches in diseases where an abnormal host response is elicited by factors other than infection—trauma, autoimmune disease, ischemia, and pancreatitis to name a few.

Our current approach to sepsis clinical research cannot accomplish the needed transition to models of sepsis that more reliably reflect the abnormal processes that we seek to target. Conventional clinical trials require the recruitment of thousands of patients, and measure activity using the crude and uninformative metric of survival. They study patients at one time point in a disease that evolves and changes over time as a result of evolution of the biologic process and the consequences of therapy and supportive care. They expose patients to a fixed dose and duration of therapy and incorporate no measures of biologic effect to titrate dose and duration. Additionally, they are driven by commercial pressures to achieve early and substantial financial success in a disease where experience has shown us that patient and deliberate science is needed.

We need to redefine critical illness to align disease taxonomy with therapeutic approaches [101]. This will be a mammoth undertaking, even more complex than the challenge faced by the UICC in the 1940s when it embarked on a global effort to stage cancer. It will require large-scale national and international collaboration, and will unfold over a timeframe measured in decades. Advances in data science and machine learning will help as we move towards a more effective means of personalizing the treatment of sepsis. The challenge is substantial, but the need is undeniable, and the momentum for forging such a collaborative effort is growing [101,102,103].

## 6. Conclusions

“Sepsis” studies as we have known them, in which therapies are administered to large, heterogeneous populations of patients with sepsis without consideration for immune status or likely response of individuals to that therapy, may no longer have a place in sepsis research. Rather, a progression toward optimizing the host response in a personalized manner is more relevant, with treatments selected based on biomarkers that reflect the pathological process, the phase of disease, and individual patient characteristics, as assessed using genomics, transcriptomics, and proteomics (Table 2).

Understanding and being able to monitor the pathways affected by any potential sepsis treatment is the key to success. Mortality will remain an important end-point, but is no longer the only one.

## Figures and Tables

**Table 1:** Some interventions targeting the hyperinflammation of sepsis that have been assessed in clinical trials, with some key references.

Intervention	Refs	Intervention	Refs
Corticosteroids	[13,14,15,16]	Alkaline phosphatase	[19,20]
Nonsteroidal anti-inflammatory agents (ibuprofen)	[18]	Statins	[21,22]
Anti-TNF (antibodies, receptors)	[5,23]	Activated protein C/thrombomodulin	[24,25,26]
Anti-IL-1 (IL-1ra)	[6,27]	TFPI/antithrombin	[28,29,30]
Anti-TLR4	[31,32]	Lactoferrin	[33,34]
Bradykinin inhibitors	[35]	Levocarnitine	[36]
Interferon	[37]	Thymosin alfa 1	[38]
Anti-PAF	[7,39]	Antioxidants (N-acetylcysteine)	[40]
Nitric oxide inhibitors/scavengers	[41,42]	Vitamins	[43]
Antiendotoxin (antibodies, purification)	[44,45,46,47,48]	Traditional Chinese medicines (e.g., Xuebijing)	[49]

TNF: tumor necrosis factor; IL: interleukin; ra: receptor antagonist; TLR: Toll-like receptor; PAF: platelet-activating factor; TFPI: tissue factor pathway inhibitor.

**Table 2:** Future evolution of “sepsis” trials.

The Past	The Future
**Preclinical studies**	
Limited data from previously healthy animals made septic (e.g., CLP) Limited information on the pathophysiologic processes	Larger variety of animal studies Better definition of the pathway of interest More information on the pathophysiologic processes Development of suitable biomarkers
**Clinical studies**	
***Patient selection*** Severe infection with some degree of organ failure	***Patient selection*** Based on the pathophysiologic process (ideally guided by a biomarker) Infection may not be required
***Treatment dose and duration*** Arbitrarily defined	***Treatment dose and duration*** Individualized (ideally guided by the biomarker)
***Primary end-point*** 28-day mortality	***Primary end-point*** Morbidity (and mortality)

CLP = cecal ligation and puncture.

## Data Availability

Not applicable.
